# The rDNA Loci—Intersections of Replication, Transcription, and Repair Pathways

**DOI:** 10.3390/ijms22031302

**Published:** 2021-01-28

**Authors:** Ivana Goffová, Jiří Fajkus

**Affiliations:** 1 Laboratory of Functional Genomics and Proteomics, National Centre for Biomolecular Research, Faculty of Science, Masaryk University, CZ-61137 Brno, Czech Republic; ivanka.goff@gmail.com; 2 Chromatin Molecular Complexes, Mendel Centre for Plant Genomics and Proteomics, Central European Institute of Technology, Masaryk University, CZ-62500 Brno, Czech Republic; 3 Department of Cell Biology and Radiobiology, Institute of Biophysics of the Czech Academy of Sciences, CZ-61265 Brno, Czech Republic

**Keywords:** rDNA organization, genome stability, rRNA genes, ribosome, CAF-1, RAD51, RTEL1

## Abstract

Genes encoding ribosomal RNA (rDNA) are essential for cell survival and are particularly sensitive to factors leading to genomic instability. Their repetitive character makes them prone to inappropriate recombinational events arising from collision of transcriptional and replication machineries, resulting in unstable rDNA copy numbers. In this review, we summarize current knowledge on the structure and organization of rDNA, its role in sensing changes in the genome, and its linkage to aging. We also review recent findings on the main factors involved in chromatin assembly and DNA repair in the maintenance of rDNA stability in the model plants *Arabidopsis thaliana* and the moss *Physcomitrella patens*, providing a view across the plant evolutionary tree.

## 1. Introduction

Translation, the rewriting of a sequence of nucleotides from mRNA into a chain of amino acids forming a protein, is an essential and fascinating process in a cell’s life as most biological activities are performed by proteins. Ribosomes are the translational machines that decode information carried by mRNA. It is not surprising that ribosomal RNA (rRNA) and proteins forming the large 60S subunit and the small 40S subunit of the eukaryotic 80S ribosome are extremely abundant, with rRNA making up over 80% of the total RNA in a cell [1]. Biogenesis of ribosomes therefore, greatly affects the rate of cellular growth and proliferation due to energy and nutrient demands [2]. To meet this enormous demand, rRNA genes (rDNA) are present as multicopy gene arrays in all eukaryotic genomes.

Because of its central role in cellular metabolism, rDNA is maintained with a high degree of evolutionary conservation. The 18S, 5.8S, and 25S (in plants) or 28S (in mammals) rRNA genes are clustered together, forming the 45S transcription unit in plants, the 47S unit in mammals, or the 35S unit in yeast [3]. Transcription of this unit to pre-rRNA is performed by RNA polymerase I (Pol I) in all eukaryotes (reviewed in [4]). Interestingly, although 18S rRNA is encoded strictly as a part of the 45S cluster, it is the only RNA molecule included in the small 40S ribosomal subunit. Genes encoding 5S rRNA, which is a part of the large 60S ribosomal subunit, together with 25S and 5.8S rRNA, are localized either in clusters separated from those of 45S rDNA (S-type arrangement), or as individual copies inserted between 45S transcription units (linked or L-type arrangement) among eukaryotes [5]. Moreover, 5S rRNA is transcribed by a different RNA polymerase—RNA polymerase III (Pol III).

rDNA loci, being repetitive sequences where transcription and replication machineries meet, are prone to recombination events that make this region one of the most unstable [6]. Events such as DNA damage or a stalled replication fork are usually repaired by homologous recombination (HR), where the neighboring repeat acts as the template. Sometimes, during such a repair event, intervening units that form a loop between the donor and acceptor sites can be excised and the number of rDNA repeats is, therefore, reduced [7]. Corresponding reverse HR events may multiply rDNA copy numbers. This makes rDNA regions, together with telomeres and other repeats, hotspots of general genomic instability.

In plants, current knowledge of rDNA loci number, copy number, and separated or linked arrangements is focused on seed plants (according to The Plant rDNA database, where bryophytes are represented by only two families [8]), reflecting the fact that rDNA within early diverging plants is still shrouded in mystery. In this review, we provide a deeper insight into the organization of rDNA in model plants, with emphasis on known differences between lower and higher flowering plant species. We also review the role of genomic instability of plant rDNA loci and mechanisms of maintaining their integrity.

## 2. Genomic Organization of Ribosomal Genes

Genes coding 45S rRNA usually form one of the most abundant gene families in eukaryotes (e.g., 10% of the whole genome in *Saccharomyces cerevisiae* [9] or 4% of the *Arabidopsis thaliana* genome [10]), with substantial individual variability in copy number and a highly homologous (homogenized) nucleotide sequence in a transcribed region coding for 18S, 5.8S, and 25S rRNA. rDNAs form one or more tandemly arranged gene clusters (nucleolus organizing regions, NORs) per haploid genome, frequently located adjacent to heterochromatic regions such as telomeres or centromeres [11]. In human cells, a total of ~350 gene copies are distributed between five acrocentric chromosomes, on their short arms [12] near the centromere and flanked by heterochromatin junctions [13]. In the yeast *S. cerevisiae*, there is just a single cluster consisting of 150 rDNA copies [9], where each rDNA unit also comprises a copy of the 5S rRNA gene positioned in the intergenic spacer, in contrast to, e.g., mammals with separate 5S gene clusters.

**Figure 1:**
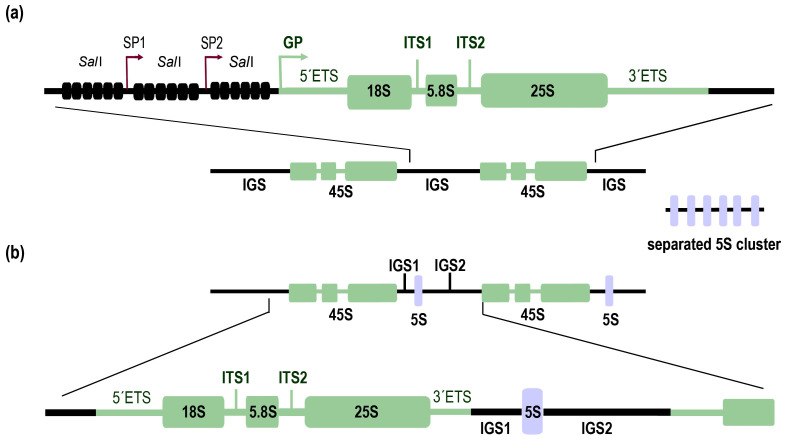
Organization of nuclear ribosomal RNA (rRNA) genes in plants. (**a**) A schematic view of rDNA organization in *Arabidopsis thaliana* S-type. A single 45S gene unit consists of three genes (18S, 5.8S, and 25S) transcribed together as a single transcript. Genes are separated by internal transcribed spacers (ITS1 and ITS2). Borders of 45S rDNA are formed by external transcribed spacers (5’ETS and 3’ETS). Adjacent 45S rDNA units are separated from each other by intergenic spacers (IGS). 5S rDNA is located at a separate locus. (**b**) A schematic view of rDNA organization in *Physcomitrella patens* L-type. 45S rDNA is organized similarly to *A. thaliana* with a different 5S rDNA position. The 5S rRNA gene is linked to 45S rDNA and is incorporated into the IGS.

Plants in general contain large numbers (from hundreds to thousands) of rDNA copies [14]. The best explored model plant, *A. thaliana,* with many ecotypes worldwide, differs in rDNA copy numbers, ranging from 500 to 2500 in the haploid state [15]. According to Rabanal et al. (2017) [16], 500 copies of rDNA can be considered as the lowest number in naturally occurring *A. thaliana*, which is still much higher than in mammals. In the frequently studied ecotype Columbia-0 (Col-0), ~750 tandem 45S rDNA genes are divided into approximately two halves in subtelomeric regions of chromosomes 2 and 4 (NOR2 and NOR4) [17]. A single 45S rDNA repeat unit (Figure 1a) contains 18S, 5.8S, and 25S rRNA genes separated by two internal transcribed spacers (ITS1 and ITS2) and two external transcribed spacers (5’ETS and 3’ETS), located at the borders of the transcribed regions [18,19,20]. Individual units are separated from each other by intergenic spacers (IGSs), also known as nontranscribed spacers (NTSs). This region has characteristic features of tandem repeats enriched with a *Sal*I restriction site (*Sal*I boxes), one gene promoter (GP), and a variable number of spacer promoters (SP1 and SP2 in the data from [21]) that share 90% sequence similarity with the GP, but achieve just 10% of GP activity [19,22,23]. The function of SPs and *Sal*I boxes is not clear. However, their sequence consists of multiple sites homologous to small interfering RNA (siRNA), suggesting their role in rDNA silencing [22].

Although rRNA sequences of *A. thaliana* are evolutionarily highly conserved, considerable variability can be seen in lengths and nucleotide sequences of intergenic spacers in both transcribed and non-transcribed regions. Extensive studies of 3’ETS revealed four 3’ETS variants distinguishing between four 45S rRNA gene subtypes, termed VAR1–VAR4 [24]. Recently, a fifth variant, VAR5, was reported by Havlová et al., [25]. The distribution of 3’ETS variants in NORs was described, showing that VAR1, representing approximately 50% of rRNA genes together with a small fraction of VAR3, is located in NOR2, which is transcriptionally inactive except during early development. The variants VAR2, VAR3, and VAR4 were then mapped to locus NOR4, which consists of transcribed genes [21]. However, even this locus is not fully transcriptionally active as the level of expression of individual variants changes during plant development. The rDNA variant VAR5, whose definition is based on the distance of the first *Sal*I box from the end of 25S rDNA and is closely related to VAR3 (differing in a 100-bp deletion at the distal site), has not yet been localized to a specific NOR. However, we can deduce its location on the basis of similarity with VAR3, which has been localized to both NOR2 and NOR4. However, a version of VAR3 with a *Hind*III restriction site (occurring also in VAR5) is localized exclusively to NOR4. A similar location is thus expected in the case of VAR5 [25]. The latest study characterizing precisely rDNA organization of NOR2 by combining short- and long-read sequencing technologies revealed non-random higher-order arrangements of rDNA variants into distinct clusters. Importantly, the study also showed that distinct rDNA variants are expressed and integrated into mature and translating ribosomes in a tissue-specific manner [26]. Remarkably, when the rRNA genes are translocated from inactive NOR2 to active NOR4, the translocated genes become active. This indicates that rRNA gene regulation based on NOR location (presumably through the more heterochromatic environment adjacent to the proximal end of the rDNA cluster in NOR2) is more important than hypothetical regulation based on sequence differences among gene variants [27]. This has also been confirmed in *Arabidopsis* plants with dysfunctional genes encoding subunits of the histone chaperone chromatin assembly factor-1 (described in detail in Section 4).

The smallest, but still essential RNA component of ribosomes that must be mentioned is the 5S rRNA molecule. A typical *Arabidopsis* 5S rRNA gene includes a 120-bp-long Pol III transcribed sequence followed by a 380-bp spacer region organized together in a multicopy gene array separated from 45S rDNA, as in mammals [28]. This separated (S-type) arrangement of rDNA units generally occurs in most angiosperms, except *Artemisia* (family Asteraceae), where linkage of 35S and 5S rRNA genes occurs [29]. Previously, the 5S rDNA copy number in the Col-0 ecotype was estimated at about 1000 copies per haploid genome [30]. However, more recent studies using next generation sequencing showed that the Col-0 genome comprises over 2000 5S rRNA gene copies. Moreover, other *Arabidopsis* ecotypes vary in 5S gene copy number over a range of 800 to 4800 copies without affecting levels of 5S transcripts [31]. Physical mapping [32] and fluorescence in situ hybridization (FISH) [33] revealed 5S localization to the pericentromeric area of chromosomes 3, 4 and 5. Even though 5S rDNA copies, as typical for ribosomal DNA, are almost identical due to concerted evolution, 5S rRNA can contain polymorphisms. Based on the presence of these polymorphisms, the major and minor 5S rRNA gene fractions are distinguishable [28]. The major genes (encoding the consensus transcribed sequence) are localized to the transcriptionally active loci at chromosomes 4L and 5L, which are enriched in transcription-permissive epigenetic marks, while the locus on chromosome 3 is the most polymorphic (minor 5S genes) and epigenetically determined as transcriptionally inactive [31,34].

Studies of rDNA organization in early land plants is complicated, particularly in the case of FISH experiments, by the usually small size of chromosomes and their apparent structural uniformity (distribution of euchromatin and heterochromatin) without distinct centromeric heterochromatin [35]. This led to the generalization of the S-type clustering for all major plant lineages. More light on this topic was brought by Sone et al. [36], who demonstrated the linked (L-type) arrangement (Figure 1b) in liverwort *Marchantia polymorpha* and the moss *Funaria hygrometrica*. Further studies led to the proposal that the physical linkage of all rRNA genes in one cluster was a general feature of early land plants. This hypothesis was strongly supported by analyses of algae, liverworts, mosses, hornworts, and lycophytes. Interestingly, conversion from the L-type to S-type arrangement was found in chlorophytic algae, where a separated 5S rDNA cluster and no residual linked rDNA was identified in the well-known model organism *Chlamydomonas rheinhardtii;* this was in contrast to other species belonging to this taxon. A similar situation was observed in monilophytes, where S-type organization was demonstrated in water ferns [5]. Recently, the previous failure of 5S cluster detection in the moss *Physcomitrella patens* [5] was clarified experimentally using PCR and FISH [37]. The impressive technique of Extended DNA Fiber FISH (EDF-FISH) clearly showed the physical association and intermingled arrangement of 5S and 5.8S rDNA on individual DNA fibers. Although EDF-FISH provides an elegant solution for fine DNA visualization in problematic chromosomes of early land plants, an effective experimental procedure to define localization of an active NOR cluster to specific chromosomal loci (*P. patens n* = 27) remains elusive. Nevertheless, FISH performed on isolated nuclei of *P. patens* displays nucleolar localizations of rDNA to three distinct foci [37]. In other species representing major clades of early land plants, the presence of rDNA chromatin knobs was restricted to just one or rarely two rDNA sites [38]. The copy number in *P. patens* was estimated to be ~900 copies [37,38] whereas in other bryophytes, similar to angiosperms, copy number ranges from hundreds to thousands of rDNA units [38]. How rRNA transcript dosage is regulated remains unclear. The epigenetic mechanisms have been studied mainly in *P. patens* and *M. polymorpha,* where, as expected, the majority of methylated cytosines are concentrated in heterochromatin [35,39]. The analyses of rDNA methylation in bryophytes revealed variation among species, ranging from negligible (<3% in *P. patens*) to moderate (7% in *M. polymorpha*) levels. These levels were lower than methylation of other repetitive sequences in the respective species [39,40], indicating that in early diverging plants, cytosine methylation does not play as important role in epigenetic regulation of rDNA, as it does in seed plants, and distinct epigenetic marks dominate the regulation of rDNA expression [40].

## 3. rDNA as a Sensor of Genomic Instability and Aging

Nuclear rDNA has long been considered to be merely involved in ribosomal biogenesis, nucleolus formation and protein synthesis. Over the past two decades however, this perception has radically changed. It has been suggested that rDNA plays important roles in preserving genomic stability, modulating gene expression and cellular aging [38]. High demands for rRNA production and the tandemly repeated nature of rDNA make the locus one of the most demanding genomic regions for stable maintenance. Instability arising from the requirement for transcriptional activity during almost the entire cell cycle, and resulting in frequent recombination events, needs special attention in order to maintain these loci.

In all eukaryotes, the rDNA copy number is much higher than is actually required for a needed dose of rRNA transcripts. In yeast, flies, plants, and also humans, only 50% of rRNA genes are transcribed [41]. For many years it was thought that this was the consequence of instability, until the outcome of many observations revealed that this high copy number is maintained during normal growth conditions. Studies focusing on the regulation of instability and maintenance of rDNA tracts in budding yeast [42,43,44] provided data to better understand the function of these extra rDNA copies. At first, it was observed that yeast cells can survive a reduction in rDNA copy number without affecting levels of rDNA transcription due to an increase in RNA Pol I loading per transcribed repeat [9]. On the other hand, Ide at al. showed the importance of redundant rDNA copies for efficient DNA damage repair as they protect sensitive budding yeast from exposure to UV irradiation and other agents causing DNA damage, and are required for proper rDNA cohesion during repair [42]. Recent studies of essential genes in yeast temperature-sensitive mutants demonstrated that mutants with affected DNA replication often had reduced rDNA arrays that allowed timely completion of DNA replication as an adaptation to replication stress [44]. Previously, it was also observed that mutations in the key replication initiation complex in yeast (origin recognition complex, ORC) could be rescued by rDNA array contraction [45], suggesting problems with the repetitive character of rRNA genes in the replication stress response. Taken together, these data suggest that the heterochromatic state of the inactive fraction of rDNA may be crucial for maintaining rDNA stability, as heterochromatic copies are less accessible to damaging factors and also reduce the transcriptional load under normal conditions. However, under stress conditions, they can become a burden, which has to be removed to facilitate whole genome maintenance. The requirement for redundant heterochromatic rRNA genes to maintain genome stability is also obvious in humans, as demonstrated by experiments with human cell lines exhibiting DNA methylation mechanisms affected by treatment with DNA methylation inhibitors or mutations inactivating DNA methyltransferases. These cell lines showed genomic instability, as manifested by an increase in the formation of extrachromosomal rDNA circles (ERCs) [46]. ERCs mainly originated from highly transcribed GC-rich sequences such as rRNA genes [47]. It was also demonstrated that ERCs are more likely to be generated by breakage events depending on an RNA/DNA hybrid or R-loop formation where a nascent RNA transcript was intercalated into the DNA duplex [48].

rDNA stability is linked with cellular lifespan in yeast through many distinct molecular pathways [49]. The accumulation of ERCs as byproducts of rDNA instability has been proposed as a major mechanism of lifespan control in yeast [50]. Indeed, the level of ERCs present in the cell correlates with lifespan; an increase in ERC production shortens yeast longevity and vice versa [51]. The maintenance of rDNA, which is critical in yeast aging, is highly associated with a function of the replication fork barrier (RFB), located downstream of the pre-rRNA coding sequence [52]. On one hand, Fob1 protein binds to the RFB and thus inhibits DNA replication, thereby avoiding a clash between transcription and replication machinery [53]. This often leads to a collapse of the replication fork and the formation of a double-strand break (DSB), resulting in a loss of rDNA copies. When cells were depleted of Fob1p, rDNA stability and lifespan increased [54,55]. On the other hand, Fob1p binding is regulated by the histone deacetylase Sir2p, via its impact on a non-coding RNAPII-dependent promoter of E-pro transcriptional activity [56]. E-pro transcriptional activity causes the removal of cohesin from sister chromatids, resulting in unequal pairing and a subsequent increase in copy number. The role of Sir2p is to repress E-pro transcription and then to promote equal sister chromatid recombination without a change in copy number [57]. Correspondingly, inactivation or mutation of Sir2 resulted in an accumulation of ERC (originating from aberrant intra-chromosomal recombination) and reduced yeast lifespan [58]. This linkage of rDNA stability and cellular lifespan may be broken by deletion of factors related to DNA polymerase ε, which leads to instability of the ribosomal tract without emission of an aging signal. Moreover, these mutations also suppress the short lifespan phenotype of *sir2* mutants, suggesting that E-pro transcription is linked to the production of an aging signal whose level is related to the number of unequal sister-chromatid recombination events [43].

Similarly to yeast, aging in mammals is also associated with rDNA instability, mostly originating from a collapse of RFBs, which were also identified in mouse and human rRNA genes [59]. It is not surprising then that such instability of ribosomal genes is manifested in human progeroid syndromes such as the Werner, Bloom and Cockayne syndrome or ataxia telangiectasia [60,61,62]. The factors whose malfunction is causal to these premature aging syndromes (WRN, BLM, CSB, and ATM, respectively), besides their other functions in DNA repair and replication, were described as interacting partners of RNA Pol I. Therefore, depletion of these genes causes down-regulation of rDNA transcription, resulting in respective disease onset (for review see [41]). Furthermore, recent evidence suggests that disruption of rDNA stability, promoting the generation of ERC and RNA:DNA hybrid molecules, can excite pro-inflammatory receptors in human cells, which is consistent with the frequent occurrence of systemic pro-inflammatory activation in patients affected by these syndromes [63]. However, not only human progeria is interconnected with rDNA instability. In contrast to progeroid syndromes, up-regulation of rRNA transcripts is often a characteristic of cancer cells due to their enormous requirements for protein synthesis to cover rapid cellular proliferation leading to particular instability in rDNA loci [64].

## 4. Factors Contributing to rDNA Stability in Plants

Plants, as sessile organisms, are continuously exposed to stress due to changing environmental conditions and, therefore, need to put particular effort into maintaining genome stability, especially at sensitive repetitive loci such as telomeres and rDNA. The huge profusion of rRNA gene copies in plants plays an important role in the maintenance of genomic stability because, under the pressure of adverse changes in various genetic or epigenetic factors, the cell reacts to the situation by a change in rDNA copy number. Among many factors affecting rDNA stability in model plants *A. thaliana* and *P. patens*, here we focus on those involved in nucleosome assembly, DNA repair, and guanine quadruplex (G4) formation and resolution.

### 4.1. Histone Chaperones

Obstacles for the replication machinery, leading to stalling of the replication fork, represent one of the major factors destabilizing rDNA loci in plants. For proper cell functioning, accurate DNA replication is needed, as well as the associated efficient propagation of chromatin structure. The dynamics of telomeric and rDNA chromatin are directly regulated by correct histone incorporation [65]. Central players in the reassembly of basic chromatin units, the nucleosomes, after their replication-coupled disruption, are histone chaperones [66]. Histone chaperones safeguard and prevent histones from inappropriate deposition onto DNA through binding of histone dimers [67]. One representative member of this protein group with an impact on rDNA stability is chromatin assembly factor-1 (CAF-1). CAF-1 is a histone chaperone, highly conserved among eukaryotes and consisting of three subunits referred to as: FASCIATA 1 and 2 (FAS1, FAS2) and MULTICOPY SUPPRESSOR of IRA 1 (MSI1) in plants; chromatin assembly complex (CAC1-3) in budding yeast; and p150, p60, and p48 in mammals [68,69,70]. CAF-1 is responsible for the formation of (H3H4)_2_ tetramers from H3H4 dimers (assembled by the chaperone anti-silencing factor 1 (ASF1)) and their loading onto DNA during DNA replication [71]. Apart from this function in replication-dependent chromatin assembly, CAF-1 is also localized to DNA damage foci for chromatin restoration upon double-strand break (DSB) repair and nucleotide excision repair (NER) [72,73,74]. In comparison to yeast, where loss of function of CAF-1 is tolerated [75], in mammals CAF1 is essential for viability [76]. In *A. thaliana* mutants lacking functional CAF-1 subunits, either FAS1 or FAS2, show serious phenotypic consequences such as abnormal morphology of flowers and leaves [68], but only the MSI1 subunit mutation is embryo-lethal [77]. In addition to morphological defects, *fas* mutants were manifested in progressive telomere shortening and loss of 45S rDNA, while the 5S rDNA cluster remained untouched. The reduction in 45S rDNA copy number is as dramatic, where in the fifth generation of mutants, only ~10% (114 genes) are left and thus all remaining genes are needed to be transcriptionally active in order to satisfy overall rRNA demand [78,79]. It was shown that active (intranucleolar) rDNA copies are lost preferentially. The lost copies are subsequently replaced with originally inactive copies (VAR1) that move to the nucleolus, become active, and then also progressively disappear. Even though replication fork stalling is severe for rDNA stability, recent studies show that the homology-dependent DNA damage repair (HDR) pathway—a single-stranded annealing (SSA) recombination—is involved in rDNA loss in CAF1-deficient plants. In this pathway, typical for direct repeats, a broken unit is cut out and adjacent repeats next to the break anneal and ligate to renew DNA integrity. This mechanism of rDNA loss was proven by testing the effect of the absence of RAD51B in a background of *fas* mutations. The knockout of *RAD51B,* which is involved in the SSA–HDR pathway, decreased the rate of 45S rDNA loss in *fas* mutants. Moreover, involvement of DNA repair in the dynamics of rDNA is supported by the increase in DSBs occurring independently of replication in 45S rRNA genes, especially in transcribed regions [80]. The fact that cell cycle-related defects and increased levels of HR were previously reported in *fas* mutants [81,82,83] only reinforces the significance of these results. Interestingly, *CAF-1* revertant lines (plants segregated as wild type (WT) plants from the crossing between *fas1* and *fas2* plants) show diverse abundance and rearrangements of rDNA variants (including their distribution between NOR2 and NOR4), and reprogramming of their activity, which then remains stable after the initial recovery phase, lasting for 2–3 generations from restoration of CAF-1 function. Intriguingly, all of these lines with mutant history showed the WT phenotype, independent of their rDNA abundance (ranging among different plant lines between ca. 20% and 150% with respect to WT plants without mutation history) [10,84]. Surprising overall phenotypic restoration of *fas* plants to WT condition was observed after deletion of three of four genes encoding the histone chaperone nucleosome assembly protein 1 (NAP1) [85]. NAP1 primarily deposits H2A–H2B histone dimers into (H3H4)_2_–DNA complexes (tetrasomes) pre-assembled by CAF-1, and function in the promotion of nucleosome assembly and disassembly during transcriptional activation [86,87]. Thus it is considered to be an H2A–H2B histone chaperone in contrast to CAF-1, although it also has the ability to bind the H3H4 tetramer in yeast and mammals [88]. The absence of NAP1 in backgrounds of *fas1* mutants suppressed the loss of 45S rDNA to 60%–80% of the WT copy number and this state was maintained across generations [85]. As NAP1 proteins were shown to be interconnected to HR [89], it is possible that the cause of rDNA copy number restoration is disruption in the HR pathway similarly to the effect of RAD51B depletion, where a partial protection of rDNA against the loss also occurred [80].

In addition to knowledge about the function of histone chaperone CAF-1 itself, plant lines generated as segregated WT plants from a cross between *fas1-4* and *fas2-4* displayed immediate resumption of the WT phenotype despite a diverse recovery of rDNA copy numbers, changed representation and reprogramming of rDNA variants and their associations with NOR2 and 4 [84]. In particular, the line containing and maintaining only 20% of rRNA genes (named as 20rDNA [90]) provides a unique plant model to study the molecular biology of rDNA in the absence of a high abundance of inactive rRNA genes. Its use as an experimental system helped to reveal the linkage between rDNA methylation and expression [79] and allowed the use of super-resolution microscopy techniques such as structure illumination microscopy (SIM), which uncovered details of cell cycle-dependent dynamics of rDNA architecture, transcription and replication [10]. Moreover, a very recent study showed that rDNA instability and copy number reduction connected with the *CAF1* mutation leads to several large tandem duplications ranging from 57 kb to 1.44 Mb. The duplicated genes increased their levels of transcription, correlated with the acquisition of increased resistance to different plant pathogens [90]. Hence, changes in copy number of rRNA genes can act as an important tool in genomic evolution.

### 4.2. RAD51 and RTEL1

Illegitimate HDR provides a magnificent source of genomic aberrations throughout the eukaryotic kingdoms. From this point of view, the link between rDNA loss and this specific DSB repair mechanism in plants is not that surprising. RAD51, the ortholog of RecA recombinase in bacteria, is a key player in the homology search and strand invasion step in error-free HR. In the *A. thaliana* genome, in addition to AtRAD51, there are five paralogues, AtRAD1B, AtRAD51C, AtRAD51D, XRCC2, and XRCC3, and they are all implicated as mediators of HR catalyzed by RAD51 recombinase [91,92,93]. The loss of function of RAD51 in *A. thaliana* does not have as severe consequences as in vertebrates, where the mutation is lethal, because, in the flowering plant, it is required for meiosis (*atrad51-1* is completely sterile), but unnecessary for vegetative development [94]. No rDNA instability was reported in *atrad51-1*, and nor does *rad51b* knock-out lead to a systematic change in 45S rDNA copy number, although its absence mitigates rDNA loss in *CAF1* mutants. This is in agreement with a proposed model of rDNA loss by this specific type of HR mechanism—single strand annealing (SSA) [80]. The control of genome stability in *P. patens* is more dependent on somatic HR [95]. Two highly homologous and remarkably intronless RAD51 genes, *RAD51-1* and *RAD51-2*, are present in the genome and their malfunction causes supersensitivity to the DSB-inducing agent bleomycin, a sensitive response to methyl methanesulfonate (MMS), and significant growth retardation [37,95,96,97]. In contrast to *Arabidopsis*, we recently reported a significant rDNA loss in the moss *P. patens* mutant with knock-out of both *RAD51* homologs. The copy number of 18S rDNA and 5S rDNA in the *pprad51-1-2* mutant dropped to 30% of WT levels, which is in line with the linked arrangement of 45S and 5S rRNA genes present in *P. patens* when the entire rDNA unit was lost, probably due to the action of the RAD51-independent repair pathway. Interestingly, the reduction in rDNA copy number did not progress in subsequent passages and the 18S rRNA transcript level remained unchanged despite the loss of a fraction of rDNA genes. This did not happen in the case of 5S rRNA transcript level, which was reduced as an indication of independently regulated transcription by RNA Pol III [37]. The antagonist of RAD51, regulator of telomere length 1 (RTEL1), is a helicase promoting a disassembly of D-loops and thus protecting the genome from inappropriate recombination [98]. RTEL1 also plays an essential role in facilitating genome replication through its interaction with PCNA [99]. Importantly, it participates in telomere maintenance as it disassembles telomeric loops (T-loops) and quadruplex structures (G4), which otherwise would block progression of the replication fork during S phase or block the extension of telomeres by telomerase [99,100,101]. In particular, the propensity for G4 formation and its resolution by RTEL1 also seems to play a role in rDNA stability. Current studies in *A. thaliana* and *P. patens* showed a reduction in rDNA copy number in plants depleted of RTEL1. In *P. patens* RTEL1 mutants (*pprtel1*), the 45S rDNA copy number, represented by 18S rDNA, moderately decreased to 75%, but as rDNA is organized in the linked arrangement between 18S-5.8S-25S units and 5S rRNA genes in this species, the 5S rDNA copy number loss is similar to the 45S gene unit. Differently from *pprad51-1-2*, transcripts of 18S rDNA were reduced accordingly to their decrease in genomic copies. The reduction in 5S rRNA transcripts was more dramatic, which again emphasizes the independence of its transcriptional regulation from the rest of the rDNA unit [37]. The loss of 45S rDNA copy numbers (to 40% of the copies in WT plants) has been observed previously, also in *A. thaliana rtel1* plants [102]. A mechanistic explanation of rDNA loss has been proposed based on the inability to resolve G4 structures with this specific helicase. This results in replication fork stalling and subsequent HR events. This mechanism has been supported in *pprtel1* plants by in silico prediction of G4 propensity, which revealed strong potential G4 sites in the spacer region between 5S and 18S rRNA genes. Surprisingly, analysis by psqfinder [103] pointed out a region ca. 500 bp upstream of the 18S rRNA gene with a score of 132 (more than double the score obtained for telomeric DNA repeat sequences) [37]. Moreover, recent analysis of the 45S rDNA unit in *A. thaliana* also showed the presence of a cluster of sites with a strong potential to form G4 structures within the gene promoter site, spacer promoters, and inside the coding regions for 18S rRNA and 25S rRNA [104].

## 5. Conclusions

Like telomeres, rDNA is an essential but also very unstable genomic element. The demand for extensive transcription of rDNA needed for ribosome biogenesis throughout the cell cycle also requires changes in the nuclear architecture that enables genome replication. The repetitive nature of rDNA generates the potential to form local specific DNA structures such as hairpins, G4 structures, or R-loops, which may initiate chromosomal rearrangements by interference with replication machinery and induction of DNA repair events. rDNA repeats are then lost, probably via ERC formation, whose accumulation is linked to lifespan in yeast and humans. In the model plants *A. thaliana* and *P. patens*, the rDNA loci are sensitive to the loss of factors involved in chromatin assembly, DNA repair, and G4 resolution. In the absence of RAD51-dependent HR, the loss of rDNA repeats can occur through the SSA mechanism. It is apparent from this review that the maintenance of such essential multi-copy interstitial chromosomal segments with unstable character requires a demanding quality check system in order to maintain its integrity and appropriate copy number.

## Data Availability

No new data were created or analyzed in this study. Data sharing is not applicable to this article.

## Abbreviations

rRNA	Ribosomal ribonucleic acid
rDNA	Ribosomal deoxyribonucleic acid
HR	Homologous recombination
S-type	Separated type
L-type	Linked type
RNA Pol I	RNA polymerase I
RNA Pol III	RNA polymerase III
Col-0	Columbia-0
NOR	Nucleolus-organizing region
VAR	Variant
ITS	Internal transcribed spacer
ETS	External transcribed spacer
IGS	Intergenic spacer
NTS	Non-transcribed spacer
GP	Gene promoter
SP	Spacer promoter
siRNA	Small interfering ribonucleic acid
FISH	Fluorescence in situ hybridization
EDF-FISH	Extended DNA fiber fluorescence in situ hybridization
PCR	Polymerase chain reaction
ORC	Origin recognition complex
ERC	Extrachromosomal rDNA circle
RFB	Replication fork barrier
DSB	Double-strand break
Fob1p	Fork blocking less protein
Sir2p	Silent information regulator 2 protein
WRN	Werner syndrome helicase
BLM	Bloom syndrome helicase
CSB	Cockayne syndrome group B protein
ATM	Ataxia telangiectasia mutated kinase
G4	Guanine quadruplex
CAF-1	Chromatin assembly factor 1
FAS1/FAS2	Fasciata 1/Fasciata 2
MSI1	Multicopy suppressor of IRA 1
CAC1-3	Chromatin assembly complex 1-3
ASF1	Anti-silencing factor 1
NER	Nucleotide excision repair
HDR	Homology-dependent DNA damage repair
SSA	Single-strand annealing
WT	Wild type
NAP1	Nucleosome assembly protein 1
SIM	Structure illumination microscopy
XRCC2/3	X-ray repair cross-complementing protein 2/3
MMS	Methyl methanesulfonate
RTEL1	Regulator of telomere length 1
PCNA	Proliferating cell nuclear antigen
